# THE FACE OF ELDER ABUSE IN AFRICA: THE NIGERIAN PERSPECTIVE

**DOI:** 10.1093/geroni/igac059.1121

**Published:** 2022-12-20

**Authors:** Eniola Cadmus

**Affiliations:** College of Medicine/ University of Ibadan, Ibadan, Oyo, Nigeria

## Abstract

Elder abuse is increasingly recognized as a serious problem in many African societies, including Nigeria. Contributing factors include a high level of poverty, unemployment, diminished availability, willingness, and ability of primary caregivers to provide for their older persons. Traditionally, elder abuse is considered taboo, and high secrecy surrounding its occurrence further contributes to challenges reporting and addressing the problem. Additionally, the patriarchal nature of the Nigerian society accounts for the gender-based differences in forms of abuse experienced with increased vulnerability among older women. This paper presents case studies that typify the experience of abuse among male and female older persons. The prevalence rates of abuse are drawn from community surveys across the six geopolitical zones. Although the methodologies vary, the total prevalence of any form of abuse experienced ranged from 30-81.1%. However, most studies relied on self-reports with no attempts at verification or data and records for triangulation of information.

